# Clinical case: Differential diagnosis of idiopathic pulmonary fibrosis

**DOI:** 10.1186/1756-0500-6-S1-S1

**Published:** 2013-04-16

**Authors:** Carlos Robalo Cordeiro, Tiago M Alfaro, Sara Freitas

**Affiliations:** 1 Centre of Pulmonology of the University of Coimbra, Portugal; 2 Department of Pulmonology, University Hospital of Coimbra, Portugal

## Abstract

**Background:**

The diagnosis of idiopathic pulmonary fibrosis can be quite challenging, even after careful clinical evaluation, imaging and pathological tests. This case report intends to demonstrate and discuss these difficulties, especially those concerning the differential diagnosis with chronic hypersensitivity pneumonitis.

**Case presentation:**

A 58-year-old white male presented with shortness of breath, dry cough, fatigue and weight loss for two months. He was a former smoker and had regular exposure to a parakeet and poultry. Physical examination revealed bilateral basal crackles and chest imaging showed subpleural cystic lesions and traction bronchiectasis with a right side and upper level predominance. Auto-antibodies and IgG immunoglobulins to parakeet and fungal proteins were negative. Lung function tests displayed moderate restriction, low diffusion capacity and resting hypoxaemia. Bronchoalveolar lavage showed increased lymphocytes (28%) and neutrophils (12%) and surgical lung biopsy was compatible with a pattern of usual interstitial pneumonia. According to the possibility of either idiopathic pulmonary fibrosis or chronic hypersensitivity pneumonitis, treatment included prednisolone, azathioprine, acetylcysteine and avoidance of contact with the parakeet, but there was an unfavorable response and the patient was subsequently referred for lung transplant.

**Conclusion:**

Chronic hypersensitivity pneumonitis and idiopathic pulmonary fibrosis can present with the same clinical and radiological manifestations In this case, despite careful evaluation, no definite diagnosis could be achieved.

## Main text

### Brief introduction

This case demonstrates the difficulties that can occur during the diagnosis of patients with Idiopathic Pulmonary Fibrosis (IPF), and the importance of careful clinical evaluation followed by the appropriate tests.

### Patient history

A 58-year old male was referred to our outpatient consultation centre with complaints of shortness of breath, dry cough and fatigue over the previous two months. He also reported anorexia and involuntary weight loss for the same period of time. His primary care physician had treated him with antibiotics, but no response or improvement in symptoms were noted. The patient’s past medical history included an episode of pesticide poisoning 35 years ago for which no information was available and occasional gout that responded to anti-inflammatory medication. The patient was an ex-smoker of 80-pack years and a moderate drinker. No known allergies were reported. His occupational history included working as a stacker in a warehouse for 20 years, with moderate dust exposure, and following this, as an administrative worker for 20 years. He was regularly exposed to a parakeet (Melopsittacus undulatus), chickens, and cats. The patient was unaware of any exposure to tuberculosis patients, recent trips abroad or family history of respiratory disease.

### Physical examination

On physical examination, he was in good general condition, but crackles were heard in both lung bases. No other changes were noted.

### Diagnostic tests

**Figure 1:**
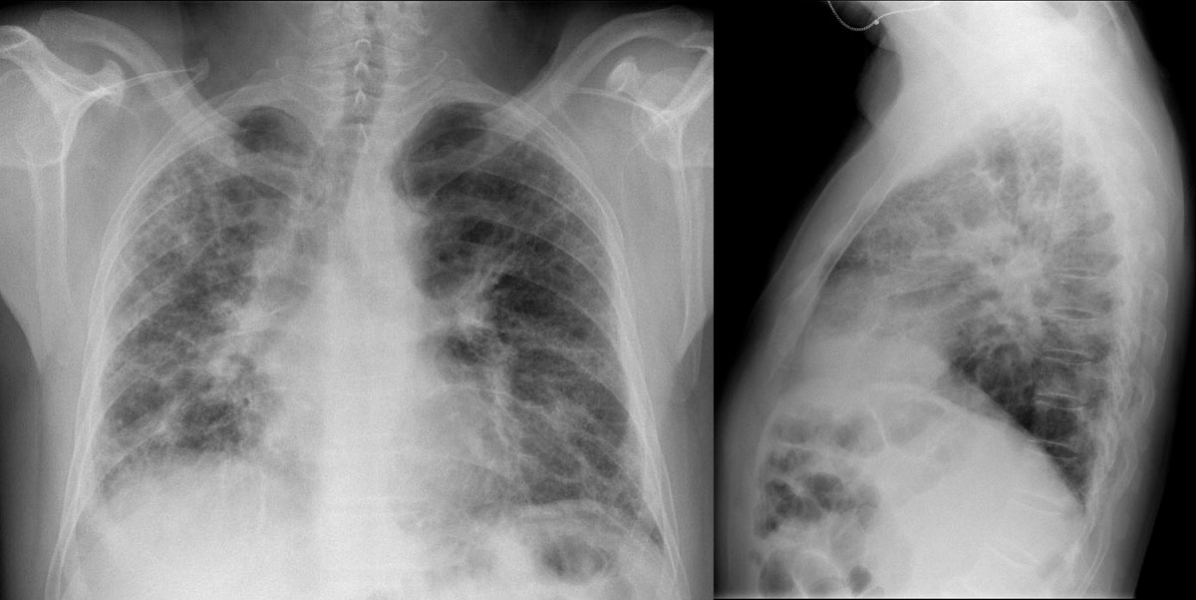
Chest X-ray showing bilateral diffuse interstitial infiltrates with a predominantly reticular pattern and no spared areas.

**Figure 2:**
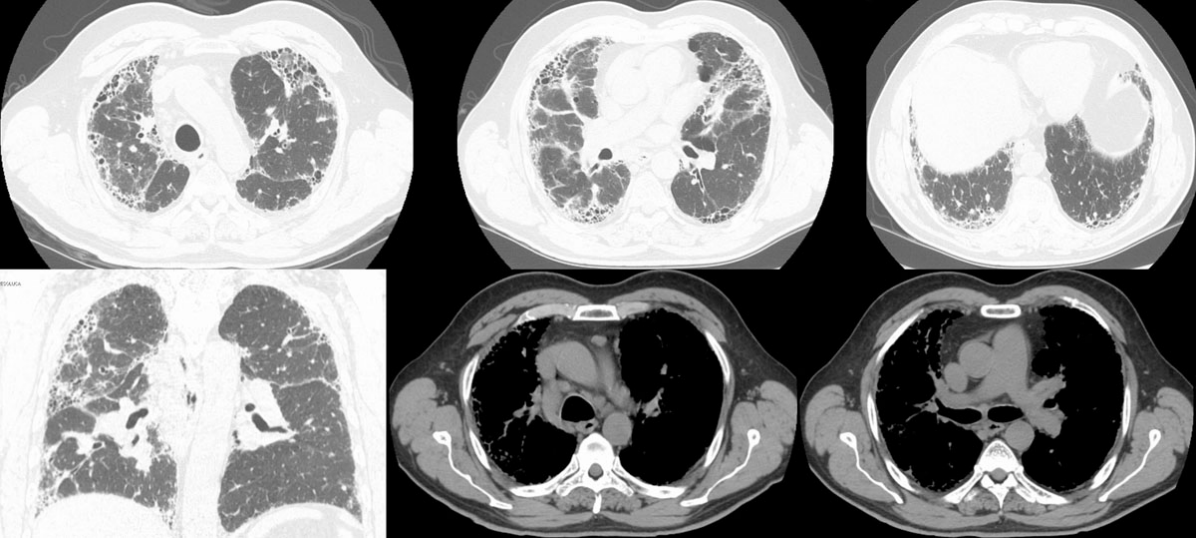
HRCT scans showing honeycombing and traction bronchiectasis affecting all lobes of the lungs, enlarged mediastinal lymph nodes and enlargement of the pulmonary artery (3.2 cm in diameter) and the right cardiac cavities.

**Figure 3:**
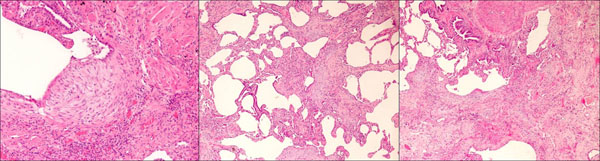
Surgical lung biopsy showing aspects compatible with a pattern of usual interstitial pneumonia.

The patient’s chest X-ray showed bilateral diffuse interstitial infiltrates with a predominant reticular pattern and no spared areas (Figure 1). This was followed by a high resolution computed tomography (HRCT) scan of the chest that showed several areas of subpleural cystic lesions and traction bronchiectasis affecting all lobes, but having an upper and middle level predominance and being much more extensive in the right lung. There were also multiple mediastinal enlarged lymph nodes and an enlargement of the pulmonary artery (3.2 cm diameter) and right cardiac cavities (Figure 2). Cardiac tests were performed, including an electrocardiogram and echocardiogram, and no other signs of cardiac disease were found. Blood tests, including those for auto-antibodies and IgG immunoglobulins (to parakeet and fungal proteins) were negative. Lung function tests suggested moderate restriction (percentage predicted forced vital capacity [FVC], 57.5%), low diffusion capacity ([DLco] 36% of the predicted value) and resting hypoxaemia (PaO2, 69.7 mmHg). The decision was made to perform bronchoscopy with bronchoalveolar lavage and transbronchial biopsy. Upon examination, the bronchial mucosa showed moderate signs of inflammation, but no other morphological changes. Bronchoalveolar lavage showed an increase in the total cell count (300 cells/µL), and increased percentage of lymphocytes (28%) and neutrophils (12%). The CD4/CD8 ratio was 0.2. Transbronchial biopsy showed no specific findings. A transthoracic biopsy was then performed, but the results were also inconclusive. The patient was referred for surgical lung biopsy. The pathology of the surgical specimen was compatible with a pattern of usual interstitial pneumonia (Figure 3).

### Treatment and patient management

At this time no definite diagnosis could be made, as the clinical, radiological and pathological findings were compatible with both chronic hypersensitivity pneumonitis and IPF. Nevertheless, treatment with prednisolone, azathioprine and acetylcysteine commenced. The patient was also instructed to avoid any contact with the parakeet. Despite the treatment, the patient got progressively worse, and has been referred for lung transplantation.

### Conclusion

Chronic hypersensitivity pneumonitis and IPF can present with the same clinical and radiological manifestations [1]. A careful clinical evaluation is therefore fundamental, and the surgical pulmonary biopsy is usually helpful in performing the differential diagnosis [2], but not in this case. A UIP pattern can be seen on biopsy (and/or CT) in both IPF and chronic HP. The addition of BAL can give a decisive contribution to the diagnostic procedures [3]. A cut-off level of 30% for lymphocytes in BAL demonstrated a favorable discriminative power for the diagnosis of IPF [4]. In this case, despite careful evaluation, no definite diagnosis could be achieved.

## Consent

Written informed consent was obtained from the patient for publication of this case report and any accompanying images. A copy of the written consent is available for review by the Editor of this journal.

## Competing interests

CRC was a speaker at the AIR meeting, receiving fees. SF and TMA reported no competing interests.

## Authors’ contributions

TMA and SF performed the data collection and drafted the manuscript. CRC conceived and supervised the whole study and made the final revision to the manuscript. All authors read and approved the final manuscript.

## Declarations

This article has been published as part of *BMC Research Notes* Volume 6 Supplement 1, 2013:IPF in 2011 – Key updates on guidelines and therapeutics. The full contents of the supplement are available online at http://www.biomedcentral.com/bmcresnotes/supplements/6/S1. This supplement originates from presentations given at the symposium “AIR: Advancing IPF Research. Working together to translate IPF research into practice” held in Berlin in November 2011. The publication was supported by InterMed Communications with funding from InterMune, AG. The content was proposed by InterMed Communications and developed with the journal. All articles in the supplement have undergone the journal’s standard peer review process.
